# Supplementary material for: Identification of a Promising Novel Genetic Source for Rice Root-Knot Nematode Resistance through Markers Associated with Trait-Specific Quantitative Trait Loci
Source: Plants (Basel). 2024 Aug 15;13(16):2271. doi: 10.3390/plants13162271 (PMC11360576; doi:10.3390/plants13162271)
Supplement: Supplementary file 1 [file plants-13-02271-s001.zip › plants-3050246-supplementary.pdf]

Table S1: Previously reported QTLs for RRKN resistance

Population	Population type	Trait mapped	QTL	Chr	Marker type	LOD	R^2	Reference
Bala × Azucena	RIL	Gall number	*qGN1*	1	SSR/RFLP	3.3	8.3	(Shrestha et al., 2007)
		Gall number	*qGN2*	2		2.9	6	
		Gall number	*qGN6*	6		4.4	9	
		Gall number	*qGN7*	7		3.2	6.5	
		Gall number	*qGN9*	9		3.6	6.5	
		Gall number	*qGN11*	11		1.49	5.2	
IR78877-208-B-1-2 × IR64	RIL	J_2RS (J_2 per root system)	*qMGR4.1*	4	SNP	3.4	5.1	Galeng- Lawilao et al.,2018)
		J_2RS	*qMGR7.1*	7		4.6	6.8	
		J_2RT (J_2 per gram of root)	*qMGR9.1*	9		3.2	4.8	
		RG (root galling)	*qRG4.1*	4		4.7	7	
		RG	*qRG8.1*	8		4.1	6.2	
		J_2RS	*qMGR$_{4.1}$*	4		4.2	6.3	
		J_2RS	*qMGR$_{7.1}$*	7		2.6	4	
		J_2RT	*qMGR$_{9.1}$*	9		3.7	5.5	
		RG	*qRG$_{4.1}$*	4		4.5	6.6	
		RG	*qRG$_{8.1}$*	8		3.5	5.2	
LD 24 × Vialone Nano and Khao Pahk Maw × Vialone Nano	F_2	Nematode resistance	*qNR11*	11	SNP			(Lahari et al., 2019)
Annapurna / Ramakrishna	RIL	Gall	*qMg-1a*	1	SSR	3.67	14.51	(Jena et al., 2013)
		Gall	*qMg-3a*	3		2.83	23.1	
		Gall	*qMg-3b*	3		2.51	11.02	

		Egg	*qMg-1b*	1		3.43	13.62	
		Egg	*qMg-3a*	3		5.03	41.1	
		Egg	*qMg-3b*	3		4.87	19.89	
Rice germplasms	GWAS	Number of galls	*qNG1a*	1	SNP			(Dimkpa et al., 2016)
		Number of galls	*qNG1b*	1				
		Number of galls	*qNG1c*	1				
		Number of galls	*qNG3.1*	3				
		Number of galls	*qNG3.1*	3				
		Number of galls	*qNG3.1*	3				
		Number of galls	*qNG3.1*	3				
		Number of galls	*qNG4.1*	4				
		Number of galls	*qNG4.2*	4				
		Number of galls	*qNG4.3*	4				
		Number of galls	*qNG4.4*	4				
		Number of galls	*qNG4.5*	4				
		Number of galls	*qNG5*	5				
		Number of galls	*qNG5*	5				
		Number of galls	*qNG11*	11				
		Number of galls	*qNG12*	12				

		Number of galls	*qNG12*	12				
		Number of galls	qNG12	12				
Rice germplasms	GWAS	Eggs/egg mass	*qEGM1*	1	SNP		9.24	(Hada et al., 2020)
		MF	*qMF1.2*	1			9.91	
		Galls, Egg mass, Eggs/ egg mass, MF	*qMF1.3*	1			9.89	
		MF	*qMF2.1*	2			12.61	
		Galls	*qG2.2*	2			7.49	
		Egg mass	*qEGG2.3*	2			8.32	
		MF	*qMF3.1*	3			10.89	
		Egg mass	*qEGG4.1*	4			7.72	
		MF	*qMF4.2*	4			6.69	
		MF	*qMF4.4*	4			10.35	
		Galls, Egg mass, Eggs/ egg mass,MF	*qMF4.5*	4			7.2	
		MF	*qMF6.1*	6			6.43	
		Galls	*qG 6.2*	6			7.32	
		MF	*qMF10*	10			5.33	
		MF	*qMF11*	11			7.13	
IR78877-208-B-1-2 and Dinorado	RIL	J_2RS	*qJ2RS*$_{3.1}$	3	SNP	4.2121	2.7386	**(Galeng-Lawilao et al., 2020**)
		J_2GRT	*qJ2GRT*$_{3.1}$	3		4.3877	2.8262	
		RG	*qGR*$_{5.1}$	5		4.0771	3.3937	
		J_2RS	*qJ2RS*$_{2.1}$	2		3.6429	2.5099	
		J_2GRT	*qJ2GRT*$_{2.1}$	2		3.49	2.5128	

		RG	$qGR_{3.1}$	3		4.5956	2.7626	
		RG	$qGR_{5.1}$	5		3.393	4.0771	
		J_2RS	$qJ_2RS_{2.1}$	2		3.5679	5.5994	
		J_2GRT	$qJ_2GRT_{2.1}$	2		2.8183	4.2386	
		RG	$qRG_{5.1}$	5		3.2977	5.0982	
PR121 (S) and *O. glaberrima* acc. IRGC102206	BC_1F_1	Number of galls	*qGN6.1*	6	SSR	3.95	41.9	(Kaur et al., 2022)
		Fresh Root Weight	*qFRW8.1*	8		3.4	17.43	
		Dry Root Weight	*qDRW3.1*	3		3.1	22.67	
		Dry Root Weight	*qDRW3.2*	3		4.8	25.4	
		Dry Shoot Weight	*qDSW3.1*	3		2.6	18.71	
		Dry Shoot Weight	*qDSW3.2*	3		4.1	21.72	
		Gall Number	*qNR2.1*	2	QTL SEQ			
		Gall Number	*qNR3.1*	3				
		Gall Number	*qNR3.2*	3				
		Gall Number	*qNR4.1*	4				
		Gall Number	*qNR6.1*	6				
		Gall Number	*qNR6.2*	6				
		Gall Number	*qNR11.1*	11				
		Gall Number	*qNR12.1*	12				
		Gall Number	*qNR12.2*	12				

Table S2. Results of single marker analysis

Chromosome	Marker Name	No of galls per plant		No of females per plant		No of egg masses/plant		No eggs/egg mass		MF	
		LOD	PVE (%)	LOD	PVE (%)	LOD	PVE (%)	LOD	PVE (%)	LOD	PVE (%)
2	RM13928	0.2256	0.6189	0.2033	0.5594	0.0454	0.1357	0.2028	0.5606	0.0764	0.2206
4	RM3735	0.535	1.4459	0.4761	1.2876	0.6451	1.7368	1.0251	2.7375	0.4723	1.2791
6	RM20158	0.0734	0.2124	1.0138	2.7084	0.5603	1.5124	0.4515	1.224	0.7405	1.9904
9	RM5122	0.6022	1.6231	1.5365	4.0652	1.5585	4.1209	1.5612	4.1307	1.9794	5.2007
10	RM25351	0.1196	0.3352	0.3165	0.8608	0.2977	0.8099	0.5976	1.6108	0.2232	0.612
11	RM6293	0.2303	0.6259	0.6024	1.6187	1.0986	2.9229	0.0657	0.1841	0.5092	1.3693
12	RM7619	0.2163	0.5964	0.333	0.9051	0.4089	1.1061	0.7131	1.9186	0.0547	0.1605
12	RM2935	0.1908	0.5256	0.3916	1.0613	0.4822	1.3024	0.2237	0.6124	0.0401	0.1193
2	RM6	0.4142	1.1231	0.2632	0.7203	0.0685	0.199	0.3275	0.8958	0.1769	0.4881
6	RM20414	0.4818	1.304	0.7393	1.9868	0.7143	1.9196	0.361	0.9842	0.9239	2.473
12	RM28165	0.4214	1.1422	0.2479	0.6765	0.1843	0.5055	0.3472	0.9461	0.3504	0.9519
2	RM263	0.0523	0.1576	0.0311	0.0963	0	0.0004	0.0462	0.1444	0.0129	0.0479
9	RM23998	1.6849	4.4501	0.4873	1.3164	0.5636	1.5186	0.2766	0.7546	0.7539	2.0207
6	RM20500	0.0643	0.1877	0.4745	1.2857	0.592	1.5983	0.1738	0.482	0.2646	0.7262
12	RM1246	0.4786	1.2943	0.2044	0.5595	0.1405	0.388	0.9389	2.5114	0.5299	1.4316
3	RM5532	0.591	1.5906	0.5079	1.3705	0.55	1.4816	0.6544	1.7592	0.6262	1.6837
9	RM23911	0.5986	1.6123	0.444	1.201	0.3881	1.0514	1.302	3.4572	1.232	3.2729
11	RM202	0.2899	0.794	0.0387	0.1175	0.1803	0.4974	0.0262	0.0814	0.0246	0.0777
2	RM6318	0.0424	0.1314	0.026	0.0831	0.0027	0.02	0.1332	0.3715	0.009	0.0354
10	RM6737	0.0435	0.1306	0.6469	1.7398	1.0702	2.8528	0.3614	0.9831	1.1338	3.0197
3	RM15652	0.8334	2.2322	0.8944	2.3912	0.7381	1.9794	1.2798	3.4021	0.8384	2.2455
3	RM15956	0.5007	1.3529	0.7064	1.8975	1.0949	2.9166	0.6454	1.7351	1.394	3.696
2	RM1234	0.0072	0.0338	0.3927	1.0648	0.2896	0.7886	0.3119	0.8488	0.3029	0.8262
11	RM552	0.4708	1.2701	1.3336	3.5394	1.0171	2.7122	0.2422	0.6639	0.8851	2.3685
11	RM3717	0.3602	0.9783	0.5305	1.4305	0.4691	1.2668	0.2616	0.7155	0.9508	2.5395
1	RM1334	0.6581	1.7735	0.1186	0.3364	0.0255	0.0845	0.9424	2.5192	0.1828	0.5054
1	RM1231	0.0819	0.2363	0.4295	1.1639	0.3457	0.9393	1.5871	4.1978	0.9133	2.4413
1	RM24	1.6445	4.3441	0.6501	1.75	0.3008	0.8211	0.1662	0.4606	0.1466	0.4087
1	RM259	0.1741	0.481	0.153	0.4251	0.4517	1.2222	0.2763	0.7576	0.1609	0.4476
